# Supplementary material for: Mitochondrial uncoupling reveals a novel therapeutic opportunity for p53-defective cancers
Source: Nat Commun. 2018 Sep 26;9:3931. doi: 10.1038/s41467-018-05805-1 (PMC6158291; doi:10.1038/s41467-018-05805-1)

Supplementary Information

Supplementary figure legends

Supplementary figure 1 Knockdown of p53 sensitizes towards niclosamide

(a) Western blot using a specific p53 antibody (DO-1) shows efficient knockdown of p53 protein expression in whole cell lysates from HCT116 cells stably transfected with p53 shI or p53 shII. (b) p53 shI sensitizes primary human fibroblasts to niclosamide. Bright field images of human primary fibroblasts stably transfected with sh vector or p53 shI after niclosamide treatment. (c) p53-independent G1 cell cycle arrest in niclosamide-treated HCT116 cells HCT116 cells stably transfected with sh vector or p53 shI. Representative flow cytometry data showing the kinetics of cell cycle progression is shown. (d) p53, p21 and MDM2 proteins detected by western blot, in untreated and nutlin (10 μM) treated ovarian cells, with differing p53 genetic status (indicated in brackets).

Supplementary figure 2 Niclosamide uncouples mitochondria independent of p53

(a) Oxygen consumption rate (OCR) was measured as a function of time (min) using an extra cellular flux analyzer (Seahorse Bioscience). Seahorse™ Mito Stress Assay was used to measure bioenergetics parameters, by adding ATP synthase inhibitor, oligomycin A. Mitochondrial uncoupling induced by FCCP using Seahorse™ Mito Stress assay. (b) Maximal respiration stimulated by the addition of niclosamide was calculated. (c) Reduction of mitochondrial membrane potential by niclosamide (2 μM) was assessed using TMRE stain. HCT116 cells were treated with niclosamide (24 hrs) followed by incubating in drug free and serum free media containing TMRE (100 nM) for 20 min. Representative flow cytometry data shown. (d) Fluorescence imaging of A549 cells treated with niclosamide (24 hrs) followed by incubation in drug free and serum free media containing TMRE (100 nM) for 20 min. Peak excitation 594 nm, emission 575 nm. Scale bars, 200 μM. (e) Quantitative analyses of cell viability (WST1) and (f) crystal violet staining of HCT116 $p53^{+/+}$ and $p53^{-/-}$ cells treated with Nic analog are shown.

Supplementary figure 3 Effects of mTOR, Wnt, STAT3 and Notch inhibitors

HCT116 $p53^{+/+}$ and $p53^{-/-}$ cells were incubated with (a-d) mTOR (e) Wnt (f) STAT3 and (g) Notch inhibitors for 24 hrs before recovery in drug free media for 4 days. Graphs show the quantitative analyses of cell viability (WST1). Data represent mean+/- SD of at least 3 independent experiments. (h) HCT116 $p53^{+/+}$ and $p53^{-/-}$ cells treated with niclosamide for 24 hrs and WCL immunoblotted using antibodies specific to mTOR, AMPK signaling proteins and (i) autophagy marker LC3BI/II.

Supplementary figure 4 Niclosamide induces global metabolic changes in cells

(a) Hierarchical clustering of differential metabolites ($p<0.05$, Welch's t-test) between niclosamide-treated and control samples derived from wild-type p53 and mutant p53 MEFs. (b) Principal component analysis scores plot describing the overall similarities and differences in the metabolic composition of wild-type and mutant p53 samples under niclosamide-treated and DMSO treated conditions. (c) Graph represents ratio of metabolites (treated over untreated conditions) for HCT116 $p53^{+/+}$ and $p53^{-/-}$ cells post niclosamide treatment. The same groups of metabolites shown for MEFs (Fig 4a) are represented here for HCT116 cells. Metabolites were measured by liquid-chromatography mass spectrometry (LC-MS). Data presented as mean +/- SD.

Supplementary figure 5 Alteration of calcium levels essential for niclosamide effects

(a,b) Calcium flux analysis of HCT116 cells treated with Nic (2 μM) using Fluo-4, AM dye in live cell imaging. Individual traces represent averages of each experiment. (c,d) Colony and cell viability data of HCT116 $p53^{+/+}$ and $p53^{-/-}$ cells treated with the indicated concentrations of FCCP for 48 hrs and recovered in drug free media for 5-8 days. (e,f) Mean values with representative images of live cell calcium imaging analyses of HCT116 $p53^{+/+}$ and $p53^{-/-}$ cells preloaded with Fluo-4, AM dye and treated with FCCP (10 μM). Averages of individual experiments are represented as individual traces in (g) and (h). (i) Metabolome analyses of HCT116 $p53^{+/+}$ and $p53^{-/-}$ cells treated with FCCP using LC-MS. Graph represents fold change (treated over untreated) of metabolite arachidonic acid (AA 20:4 ω-6) in HCT116 $p53^{+/+}$ and $p53^{-/-}$ cells. (j) Colony assay using crystal violet staining of HCT116 $p53^{-/-}$ cells treated with PLA2 inhibitors (PLA2inh I or PLA2inh II), or untreated as control. (k) Measurement of calcium levels using Fluo-4, AM dye and flow cytometry in niclosamide (2 μM)-treated cells pre-exposed to (i) calcium chelator, BAPTA-AM, only or (ii) together with EGTA (1 mM). (l) Detection of PARP1 cleavage in cells treated only with niclosamide or preloaded with BAPTA-AM (10 μM) before niclosamide treatment (+BAPTA). Densitometric measurements of cleaved PARP1/total PARP1 of a representative experiment shown in graph. (m) Apoptotic cells detected using Annexin V-FITC assay (Roche). Pre-loading of BAPTA (10 μM) alone or in combination with EGTA (1 mM) reduced niclosamide-induced apoptosis. (n) Co-treatment with carbacyclin rescued the colony growth of HCT116 $p53^{+/+}$ and $p53^{-/-}$ cells treated with niclosamide (2 μM). Untreated and niclosamide treatment alone are included. (o) WST1 cell viability data and (p) colony assay for HCT116 $p53^{+/+}$ and $p53^{-/-}$ cells treated with the indicated concentrations of carbacyclin. Data represent mean+/- SD of at least 3 independent experiments.

Supplementary figure 6 p53-dependent induction of ALOX5 and ALOX12B

(a) Efficient knockdown of p53 in A549 cells shown by western blot of WCL. (b) Relative transcript levels of a selected cohort of genes that metabolize arachidonic acid in wild-type HCT116 cells treated with doxorubicin (24 hrs). Error bars represent +/-SD of at least 3 independent experiments. (c) Detection of ALOX5 (left panel) or ALOX12B (right panel) proteins by immunoblotting of whole cell lysates harvested from HCT116 cells treated with Nutlin (10 μM) for 24 hrs or 48 hrs; untreated included as control. (d) Relative transcript levels of p53 canonical genes, *p21*, *Mdm2*, and *Alox5, Alox12b* measured by qPCR using kidney tissue-derived RNA from littermates wild-type and p53R172H mice, or from (e) littermates wild-type and p53KO mice. (f) Histogram traces of p53 ChIPseq data at the *ALOX5* and *ALOX12B* gene locus. (g) ChIP-qPCR analyses of p53 binding to the indicated genomic regions on *ALOX5* and *ALOX12B* gene locus as shown in histogram or to irrelevant regions of “A”, “B” and “C”. Data represent mean +/-SD of at least 3 independent experiments. (h-n) Gene profiling in ovarian samples using large scale RNA-seq trancriptomics data. Samples were ranked accordingly using gene expression profiling based on the p53 gene signature (13-gene set; *AEN, BAX, CCNG1, CDKN1A, DDB2, FDXR, MDM2, RPS27L, RRM2B, SESN1, TNFRSF108, XPC* and *ZMAT3*). Normalized $\log_2$ values from samples in the upper quartile (high expressing) and lower quartile (low expressing) groups were shown in a heat map for the 13-gene set and *ALOX5* and *ALOX21B* genes. (h) TCGA OV (ovarian) (n=216) (i) colon adenocarcinoma (COAD) (n=254). (j) Kidney renal clear cell carcinoma (KIRC) (n=274). (k) Lung adenocarcinoma (LUAD) (n=272). (l) Prostate adenocarcinoma (PRAD) (n=254). (m) Pancreatic adenocarcinoma (PAAD) (n=90). (n) Testicular germ cell cancer (TGCT) (n=78).

Supplementary figure 7 Silencing of ALOX5 and ALOX12B in HCT116 $p53^{+/+}$ and $p53^{-/-}$ cells. Colony assay of HCT116 $p53^{+/+}$ and $p53^{-/-}$ cells treated with niclosamide post-silencing with *ALOX5* or *ALOX12B*-specific Dhamarcon SMARTpool siRNAs.

Supplementary figure 8 Effects of niclosamide on mouse xenografts

(a) Kaplan Meier analyses of the tumor growth inhibition data presented in Figure 8a and (b) Figure 8b. Endpoint is taken as time taken for tumors to reach 800 mm^3. Statistical analysis performed using log-rank t test. (c) Immunohistochemistry of HCT116 $p53^{+/+}$ and $p53^{-/-}$ tumor xenograft sections dissected at the tumor growth inhibition study using antibodies specific against p53 protein and apoptotic marker cleaved caspase 3. (d) Immunohistochemistry using antibodies specific against the proliferative marker Ki67, p53 protein, and apoptotic marker cleaved caspase 3. Scale bars 100 μM.

Supplementary tables

Supplementary Table 1

Sensitizers of p53KO cells		Sensitizers of p53WT cells	
**compound name**	**Log2**	**compound name**	**Log2**
**NICLOSAMIDE**	**2.24**	MERBROMIN	-8.62
PROBUCOL	1.90	FLUORESCEIN	-7.06
MEBENDAZOLE	1.61	FLUMETHASONE	-3.69
OUABAIN	1.18	NAPROXEN(+)	-3.31
PROSCILLARIDIN	1.03	MITOTANE	-2.50
CEPHARANTHINE	1.02	DOXORUBICIN	-2.10
CYCLOHEXIMIDE	0.82	DAUNORUBICIN	-2.01
ROXARSONE	0.82	FLUOROURACIL	-1.82
CYCLOPHOSPHAMIDE	0.80	GEFITINIB	-1.71
BISACODYL	0.78	ACETYLCHOLINE CHLORIDE	-1.64
OXIBENDAZOLE	0.76	FLUVASTATIN	-1.56
ARSENIC TRIOXIDE	0.74	DEFEROXAMINE MESYLATE	-1.47
CEPHRADINE	0.72	PYRITHIONE ZINC	-1.37
TROCLOSENE POTASSIUM	0.70	BUCLADESINE	-1.30
ESTRIOL	0.69	CYTARABINE	-1.22
DIGOXIN	0.69	ANCITABINE HYDROCHLORIDE	-1.21
BENZBROMARONE	0.69	MYCOPHENOLATE MOFETIL	-1.10
NIFEDIPINE	0.68	CETYLPYRIDINIUM CHLORIDE	-0.93
SULINDAC	0.67	PITAVASTATIN CALCIUM	-0.89
TERCONAZOLE	0.67	VINCRISTINE SULFATE	-0.89
METHYLENE BLUE	0.66	MELPHALAN	-0.86
HEXACHLOROPHENE	0.65	EPIRUBICIN HYDROCHLORIDE	-0.82
MODALINE SULFATE	0.65	BENZOXIQUINE	-0.78
FLUMEQUINE	0.65	CICLOPIROX OLAMINE	-0.73
CYCLOPENTOLATE HYDROCHLORIDE	0.63	CARMOFUR	-0.69
CANRENOIC ACID, POTASSIUM SALT	0.63	DISULFIRAM	-0.69
RANITIDINE HYDROCHLORIDE	0.63	EZETIMIBE	-0.68
CHLOROXYLENOL	0.61	PYRVINIUM PAMOATE	-0.68
SALICYL ALCOHOL	0.61	CLOFARABINE	-0.66
SUCCINYLSULFATHIAZOLE	0.61	ATRACURIUM BESYLATE	-0.61
TILORONE	0.60	METACETAMOL	-0.60
N-METHYL (-)EPHEDRINE [1R,2S]	0.59	DECOQUINATE	-0.60
FLUTICASONE PROPIONATE	0.59	ISOXICAM	-0.58
DIFLUNISAL	0.59	AMSACRINE	-0.57
TICLOPIDINE HYDROCHLORIDE	0.59	MYCOPHENOLIC ACID	-0.56
HYMECHROME	0.58	FLOXURIDINE	-0.55
CORTISONE ACETATE	0.58	PACLITAXEL	-0.54
NEFAZODONE HYDROCHLORIDE	0.57	DENATONIUM BENZOATE	-0.52
PERICIAZINE	0.56	SELAMECTIN	-0.51
PRIMIDONE	0.56	FUMARIC ACID	-0.50
ZINC UNDECYLENATE	0.56	DASATINIB	-0.49
BENZOCLIDINE	0.55	OXEDRINE	-0.49
URSODIOL	0.55	TRIFLUPROMAZINE HYDROCHLORIDE	-0.48
CAMYLOFINE DIHYDROCHLORIDE	0.55	MEVASTATIN	-0.48
RAMIPRIL	0.54	ENOXACIN	-0.47
OXYPHENBUTAZONE	0.54	FLUVOXAMINE MALEATE	-0.47
CHLORPROMAZINE	0.54	DACTINOMYCIN	-0.46
GUAIFENESIN	0.54	BERBERINE CHLORIDE	-0.46
RASAGILINE	0.53	PIPENZOLATE BROMIDE	-0.45

HTS screening: Log_2 values of normalized ratio GFP/RFP over DMSO controls)

Supplementary Table 2

	p53$^{+/+}$				p53$^{-/-}$			
	UT		T(Dox)		UT		T(Dox)	
	**Mean**	**SD**	**Mean**	**SD**	**Mean**	**SD**	**Mean**	**SD**
p21	1.43	±0.29	14.71	±3.04	1.22	±0.23	3.32	0.61
ALOX5	3192.34	±578.08	13560.82	±294.94	402.99	±43.75	368.07	±20.17
ALOX12B	150.77	±14.53	861.31	±92.41	34.86	±0.14	29.52	±0.02
ALOXE3	628.29	±14.29	838.00	±88.80	735.06	±0.07	627.14	±14.78
ALOX12	5.90	±0.63	3.03	±0.45	4.38	±0.20	1.31	±0.43
ALOX15	1335.12	±149.13	1114.23	±428.99	1096.27	±415.46	1289.31	±195.83
ALOX15B	47.04	±8.23	50.66	±9.72	48.75	±3.84	41.17	±0.15
PTGS1	103.08	±6.06	109.26	±4.07	96.40	±4.33	88.79	±2.35
PTGS2	3.35	±0.09	1.34	±0.48	3.40	±0.46	4.57	±1.17
CYP2E1	1.38	±0.54	2.69	±0.02	3.10	±0.52	1.46	±0.13
CYP2J2	1.38	±0.54	2.69	±0.02	3.10	±0.52	1.46	±0.13
CYP2C8	2.35	±0.49	1.93	±0.11	2.38	±0.10	2.36	±0.04
CYP2C18	1.49	±0.12	1.65	±0.02	1.03	±0.04	1.51	±0.07
CYP2C19	157.25	±37.11	233.10	±35.29	70.86	±22.85	153.48	±15.65
CYP1A1	4836.72	±635.40	1858.60	±189.63	5005.96	±389.33	5120.18	±584.53
CYP1A2	57.42	±13.73	25.79	±1.19	35.14	±1.13	123.66	±20.19
CYP2S1	25.87	±1.12	18.48	±7.71	19.02	±0.72	25.68	±0.36
CYP4F8	3.07	±0.17	12.33	±5.31	4.43	±4.85	14.92	±4.79
CYP4F12	1352.14	±74.80	1723.58	±319.19	2499.12	±582.05	3117.58	±370.98

Normalized qPCR values for gene expression levels in HCT116 p53$^{+/+}$ and p53$^{-/-}$ cells. Graphs represented in Fig. 6a.

Supplementary Table 3(i)

KKH02	
Specimen type	Right and Left Ovaries
Diagnosis	Carcinoma, present both ovaries
Pathological stage	FIGO III C
Tumour size	Right ovary 5 x 6 x 1.6 cm. Cystic with a papillary area 2 x 1 cm
Gross appearance	Right : Cystic with a papillary area within it
Capsule	Left : Tumour nodule on surface<br>Right : not involved<br>Left : no capsule, tumour on surface
Location of tumour	Right : within cyst and in ovarian parenchyma<br>Left : within ovarian parenchyma and on surface
Histological type	Serous carcinoma
Grade	3; High grade
Surface Involvement	Right : No<br>Left : Yes
Adnexal tissue involvement	Right : involved – right hilar tissue and serosal surfaces<br>Left : not involved

Supplementary Table 3(ii)

KKH011	
Specimen type	Endometrial tumour
Diagnosis	Endometrioid adenocarcinoma
Pathological stage	FIGO III A
Tumour size	5 x 5.2 x 2.7 cm
Gross appearance	Fleshy, polypoidal
Capsule	Serosal involvement present
Location of tumour	Endometrial cavity
Histological type	Endometrioid carcinoma
Grade	3
Surface Involvement	Serosal involvement
Adnexal tissue involvement	Left paratubal tissue

Supplementary Table 4(i)

shRNA	shRNA constructs	Catalogue no.
p53 shRNA (I)	plko.1 shp53	Addgene Plasmid #19119
p53 shRNA (II)	plko.1 p53-shRNA-941	Addgene Plasmid #25637
p53 shRNA (III)	Plko.1 p53-shRNA-427	Addgene Plasmid #25636
Scrambled shRNA	none	Addgene Plasmid #1864

Supplementary Table 4(ii)

siRNAs	Dharma con Cat. no	Ref. sequences
Alox V (I)	J-004530-06-0010	UGAACGAUGUCUACGUGUA
Alox V (II)	J-004530-07-0010	CCAUCUGCUUGCUGUAUAA
Alox12B (II)	J-009025-06-0010	GAUACACCGUCCAGAUCAA
Alox12B (IV)	J-009025-08-005	GCACGCGGAUCCCAGACAA
Luc	# D-002050-01-20	CGUACGCGGAAUACUUCGA
Alox12B(smart pool)	L-009025-0005	ON –TARGET plus Human ALOX12B
Alox 5(smart pool)	L004530-00-0005	ON-TARGET plus Human ALOX5

Supplementary Table 5 (i)

Target gene	Forward (5'- 3') primers	Reverse (5'- 3') primers
Alox12B(M)	GCGAACTATACCTGAGCTGAT	GTAGTTAGCCCCTTGGTCTG
Alox12B(M)	TGGGGGAGCTTATCATCATC	GAGTGCCAGGGTCTCATAGC
AloxV(M)	GACTACATCGAGTTCCCATGTTA	GTG CTGCTTGAGGATGTGAATT
p-21(M)	GTC GCT GTC TTG CAC TCT GGT	GCA GAA GAC CAA TCT GCG CTT G
MDM2(M)	GCCCTCGCATCAGGATCTTG	AGCTCGTGCCCTTCGTCACT
Actin (M)	CGGTTCCGATGCCCTGAGGCTCTT	CGTCACACTTCATGATGGAATTGA
P53(M)	CCGAAGACTGGATGACTGCC	GCAGTGAGGTGATGGCAGGA
p53_Exon4 (Hu)	CACTTGTGCCCTGATTT	CTGGGGACCCTGGGCAA
p53_Exon5(Hu)	TGCTCAGATAGCGATGGTGAG	GCACATCTCATGGGGTTATAGGGA
p53_Exon6(Hu)	GGCGACAGAGCGATTCCA	GGGTCAGAGGCAAGCAGAGG
p53_Exon7-8(Hu)	GACCAAGGGTGGTTGGGAGTAGATG	GCAAGGAAAGGTGATAAAAGTGAA
p53_Exon9(Hu)	GCTGTATAGGTACTTGAAGTGCA	GAGAATGGAATCCTAGGATTTC
PTGS1(Hu)	CTT GAC CGC TAC CAG TGT GA	GTG AGT GAG CAG GAA GTG GG
PTGS2(Hu)	CTC CTG TGC CTG ATG ATT GC	CTG ATG CGT GAA GTG CTG GG
ALOX15	CGT CAT TCT CTG TAG CCA GCA	GGA AGT CAG AGC TGC GCA C
p21(Hu)	TGTAGAGCGGGCCTTTGAGG	GAAAACGGCG GCAGACCAGC
PUMA(Hu)	ACGACCTCAACGCACAGTACG	TCCCATGATGAGATTGTACAGGAC
ALOXE3(Hu)	ACT CAG CCA CCA AGA CGA TC	GCATGTCATCCAGCTTCTTCC
ALOX15B(Hu)	GGA TCT TCA ACT TCC GGA GG	GATCAGGACAGGGTTGAGAC
CYP1A1(Hu)	GCTACCTACCCAACCCTTCC	CAGCTCCAAAGAGGTCCAAG'
CYP1A2(Hu)	CCTTCAACCAGAGGTTCCTG	TCTTCTCCTGTGGGATGAGG
CYP2S1(Hu)	TACCACTGCTGGGAAACCTC	GGTCCCAGGTAGATGGTGAA
CYP4F8(Hu)	GCCTGGACCTATGCCTTCTA	GTGGCACAAGTTGATGATGG
CYP4F12(Hu)	CATCCTGGCTTGGACCTATG	GAATAGGTGGCCGACATCTG
CYP2E1(Hu)	GCA CAG CAG CTG GAA TCT G	CAC GTA CAG CGT GAA CAC C
CYP2J2(Hu)	GGC AAC TTC TTC CTT GTG GAC	GCA GAT ATG TCA CCA AGC TCC A
CYP2C8(Hu)	GCA TCA CTG GAT GTT AAC AAT CC	GAT CAG CTA CAG TGC CAA CCA
CYP2C19 (Hu)	CAG ATA GAT ATT AAG GAT GTC AGC	CAG AGT GAA CAC AGG GCC ATA
CYP2C18(Hu)	CCT GCA GTT AGA TGT TAA GGA CA	GCA ACA CCA CAA TGG GCT TCA
ALOX12B (Hu)	ACG GCC GTA TCT ACC ACT TC	AGTCCTGCTTGGCTCTGATC
ALOX12B (Hu)	GCTGAACCACTTTGGGAGAG	AGCCATCCATCCACTGGTAG
P53 (Hu)	TTC ACC CTT CAG ATC CGT GG	CAG CTC TCG GAA CAT CTC GAA
RPLO	CAGATTGGCTACCCAACTGTT	GGGAAGGTGTAATCCGTCTCC
GADPH	TGCACCACCAACTGCTTAGC	GGCATGGACTGTGGTCATGAG

Supplementary Table 5 (ii)

Peak Region	qPCR primers
(ALOX5)- p53 Peak	Forward primer: 5'-TGTGTGACTTGGGACAGCTG-3'<br>Reverse primer: 5'- TGTGTGCTAGGGGCTTTACC- 3'
(ALOX5) - "A"	Forward primer: 5'- AACCTTCTCCACACCCTTCC-3'<br>Reverse primer: 5'- AAGAGCGACCCATGAGAGAA- 3'
(ALOX5) - "B"	Forward primer: 5'- CTGTCGGAAACGGAGACTTC-3'<br>Reverse primer: 5'- ATTTACGGAGCGCTTCCTCT- 3'
(ALOX12B)- p53 Peak I	Forward primer: 5'- GGCACAGAGTCTGGAAGGAG-3'<br>Reverse primer: 5'- CCAGTCGCTGAGATGGACTC- 3'
(ALOX12B)- p53 Peak II	Forward primer: 5'-TGCCTGCTGCACTTTGGAC-3'<br>Reverse primer: 5'- GATGGCGATGGGCATCATC - 3'
(ALOX12B) - "C"	Forward primer: 5'-CTGACCCTAACTTGGCCTGG-3'<br>Reverse primer: 5'-AATAAGCGTGGGAGGTTGGG- 3'

Supplementary methods

Cell culture and reagents

HCT116 $p53^{+/+}$ and $p53^{-/-}$ cell lines (kind gifts from Dr Bert Vogelstein (John Hopkins University School of Medicine, Baltimore, MD) were grown in McCoy's 5A media, supplemented with 10% fetal bovine serum, 1% penicillin/streptomycin and 2 mM glutamine. A549 lung adenocarcinoma (ATCC CCL-185), primary human skin fibroblast and mouse embryonic fibroblasts were grown in DMEM high glucose medium supplemented with 10% fetal bovine serum and 1% penicillin/streptomycin and 2 mM glutamine. Cells transduced with lentiviral vector constructs were grown in media containing 1 µg/ml puromycin.

Chemicals and antibodies

Doxorubicin (S1208), Teniposide (S1787), stattic (S7024), Notch inhibitor (S1594), Wnt inhibitor (S1180), Wnt inhibitor 2 (S2662) and Everolimus (S1120) were from Selleck. Niclosamide (N3510), Carbonyl cyanide 4-(trifluoromethoxy)phenylhydrazone (FCCP) (C2920), 1-Methyl-2-pyrrolidinone (43729), Poly ethylene glycol 300 (202371) and Stat3 inhibitor (S3I201) from Sigma Aldrich. cPLA2α Inhibitor (525143) and cPLA2α Inhibitor II Pyrrophenone (530538) from Calbiochem, Torin I (4247), Torin II (4248), Rapamycin (S1039), Tocrisolve^{TM}100 (1684) and arachidonic acid (in Tocrisolve^{TM}100) (2756) from Tocris. BAPTA-AM (B6769) from

ThermoFisher. Carbacyclin (Carbocyclic PGI2) (sc-201250) from Santa Cruz Biotechnology. Oligomycin, FCCP, rotenone, and antimycin A, from Seahorse Bioscience.

Antibodies list

Antibody	Origin	Source	Dilution	# catalog
p53 (DO-1)	Mouse	Santa Cruz Biotechnology	1:2000	Sc-126
γ-H2AX	Mouse	Millipore	1:1000	05-636
β-Actin	Mouse	Sigma	1:5000	A1978
Hsp90	Mouse	BD Transduction	1:5000	610418
(p)-AKT-S473	Rabbit	Cell Signaling	1:1000	4060
(T)-AKT	Rabbit	Cell Signaling	1:1000	9272
(p)-AMPKα-T172	Rabbit	Cell Signaling	1:1000	2535
(T)- AMPKα	Mouse	Cell Signaling	1:1000	2793
ACC2	Rabbit	Cell Signaling	1:1000	8578
(p)-ACC-S79	Rabbit	Cell Signaling	1:1000	3661
(T)-ACC	Rabbit	Cell Signaling	1:1000	3676
(p)-ERK1/2	Rabbit	Cell Signaling	1:1000	2855
(T)-ERK1/2	Mouse	Cell Signaling	1:1000	4696
(p)-4E-BP1-T37/46	Rabbit	Cell Signaling	1:1000	2855
(T)-4E-BP1	Rabbit	Cell Signaling	1:1000	9452
(p)-p70S6K-T389	Rabbit	Cell Signaling	1:1000	9234
(T)-p70 S6K	Rabbit	Cell Signaling	1:1000	A300-510A
(p)-GSK-3β	Rabbit	Cell Signaling	1:1000	5558
Cas-3 (Asp175)	Rabbit	Cell Signaling	1:500	9661
Cas-9	Rabbit	Cell Signaling	1:500	9502
PARP1	Rabbit	Cell Signaling	1:1000	9542
ALOX12B	Rabbit	Atlas	1:500	HPA024002
ALOX5	Rabbit	BD Transduction	1:1000	610694
LC3B	Rabbit	Sigma	1:1000	L7543

Secondary screen to validate potential drug compounds

2000 cells each of H2B-GFP-HCT116p53$^{+/+}$ and H2B-RFP-HCT116p53$^{-/-}$ were grown as co-culture in 96-well plates in a total volume of 100 μl. The next day, these cells were either DMSO treated or treated with different concentrations of indicated drug compounds. After 48 hrs of drug incubation, the cell media was changed to start recovery. After 6 days of recovery, the cells were

imaged in an In Cell Analyzer 2000 machine and the percentage of viable cells was determined and plotted against the drug concentrations used in the experiment.

Niclosamide sensitivity analysis in isogenic cell lines

In another experiment, negative control scrambled shRNA (Addgene Plasmid #1864), p53-targeting pLKO-p53-shRNA-941 (Addgene Plasmid #25637) and p53-targeting shp53 pLKO.1 puro (Addgene Plasmid #19119) were used to produce different lentiviruses, each containing one of the shRNA constructs listed previously. Using the HCT116P53$^{+/+}$ cell line, cells were infected either with a control virus containing the control-scrambled shRNA or with two different viruses targeting the p53 expression. In some cases, the HCT116 cell line was infected with two independent shRNA constructs, each separately targeting p53 expression. These independent p53 expression targeting shRNA were named shRNA (I) and shRNA (II), accordingly. The one or more viruses were used at a multiplicity of infection of 2 (MOI 2) in a hexadimethrine bromide (polybrene)-mediated viral transduction. After 18 hrs of infection, the culture media was changed. After two days of infection, cells were added with cell culture media containing puromycin (1 µg/ml). After two weeks, the newly formed antibiotic-resistant clones were trypsinized and transferred into new cell culture dish with puromycin. These cells were tested for p53 depletion by Western blot analysis. Detection of a p53-knockdown of up to 90% was detected by immunoblotting.

Liquid chromatography-mass spectrometry (LC-MS) analysis

Untargeted LC-MS analysis of the polar metabolites was performed using an ultra-high performance liquid chromatography (UPLC) system (Acquity; Waters, MA, USA) coupled to a mass spectrometer (MS; LTQ-Orbitrap; Thermo Scientific, MA, USA). A reversed phase (C18) UPLC column with polar end-capping (Acquity UPLC HSS T3 column, 2.1 mm, 100 mm, 1.8 mm; Waters) was used with two solvents: 'A' being water with 0.1% formic acid (Fluka, Sigma-Aldrich, MO, USA), and 'B' being methanol (Optima grade, Fisher Scientific, PA, USA) with 0.1% formic acid. The LC program was as follows: the column was first equilibrated for 0.5 min at 0.1% B. The gradient was then increased from 0.1% B to 50% B over 8 min before being held at 98% B for 3 min. The column was washed for a further 3 min with 98% acetonitrile (Optima grade, Fisher Scientific) with 0.1% formic acid and finally equilibrated with 0.1% B for 1.5 min. The solvent flow rate was set at 400 mL/min; a column temperature of 30°C was used. The eluent from the UPLC system was directed into the MS. Electrospray ionization (ESI) was conducted in both positive and negative modes in full scan with a mass range of 80 to 1,000 m/z at a resolution of 15,000. Sheath and auxiliary gas flow was set at 40.0 and 15.0 (arbitrary units) respectively, with a capillary temperature of 400°C. The ESI source and capillary voltages were 4.5 kV and 40 V respectively, for positive mode ionization, and 2.8 kV and 15 V respectively, for negative mode

ionization. Mass calibration was performed using standard LTQ-Orbitrap calibration solution (Fisher Scientific) prior to injection of the samples.

Untargeted LC/MS analysis of the lipid metabolites was performed using a UPLC system (Acquity; Waters) interfaced with a quadrupole-time-of-flight (QToF) mass spectrometer (Xevo G2 QToF, Waters). The LC set-up consisted of an Acquity CSH (C18) column (1.0 mm × 50.0 mm, 1.7 mm, Waters) with two solvents: 'A' being methanol: acetonitrile: water mixture (2:2:1) (Fisher Scientific, Optima grade), with 0.1% acetic acid (Merck) and 0.1% ammonia (AnalaR NORMAPUR grade, VWR, PA, USA), and 'B' being isopropanol (Optima grade, Fisher Scientific) with 0.1% acetic acid and 0.1% ammonia. The LC program was set at 1% B 1 min, 1–82.5% B 10 min, 99% B 5 min, 1% B 2.2 min at 45°C and 0.1 ml/min flow rate. The eluent from the LC system was directed into the MS. Electrospray ionization (ESI) in the MS was conducted in both positive and negative modes in full scan with a mass range of 100 to 1800 m/z. The source temperature and desolvation temperature were set at 120°C and 600°C respectively while the cone gas flow and desolvation gas flow were fixed at 40 L/h and 600 L/h respectively. The lock mass compound was leucine enkephalin (m/z 556.2771 for positive mode, m/z 554.2615 for negative mode). The capillary voltage was 2.0 kV for the positive ESI mode and 1.0 kV for the negative ESI mode.

Colony forming assay

Cell viability after niclosamide treatment was analysed by colony forming assay. Briefly, 20000 cells were plated in 6-well cell culture plates with 2 ml cell culture media. The next day, cells were treated either with DMSO as a negative control or with one of five different concentrations of the niclosamide, 0.5 µM, 1 µM, 1.5 µM, 2 µM and 3 µM. After 48 hrs of drug incubation, the drug containing media was replaced with new cell culture medium and the cells were grown for another 7 days. On day 8th of the recovery, cells were stained with crystal violet and glutaraldehyde solution for 2 hrs and de-stained in plain water.

Proliferation Assay (WST1)

Cells were seeded at 1000 cell density in 96-well plate. Next day, either added with DMSO or treated with different doses of indicated compounds and incubated for 48 hrs. Cells were recovered for 6 days in fresh media. Survival assay was performed using cell proliferation WST1 reagent (ROCHE) and the wavelength absorbance was measured at 440 nm with SpectraMax M5 Multi-Mode (Molecular Devices).

Calcium measurements using Fluo-4, AM (Flow cytometry)

Cells were trypsinized and then centrifuged at 400 g for 3 min. Fluo-4, AM (Thermo Fisher Scientific) was reconstituted into 1 mM stock, and diluted into 2 µM using media. 500 µl of the

Fluo-4, AM solution was added to each cell pellet, and incubated at 37°C for 30 min. Cells were centrifuged and resuspended in PBS for flow cytometry analysis.

For BAPTA loading, cells were rinsed with 1 ml of PBS with 2 mM EDTA, before adding 2 ml of calcium-free DMEM (Gibco) containing 10 μM BAPTA-AM (Thermo Fisher Scientific). After 30 min of incubation at 37°C, the cells were then incubated with calcium-free DMEM without BAPTA-AM for 20 min to allow de-esterification. In some conditions, niclosamide (Sigma) and EGTA stock solutions were diluted in calcium-free DMEM and added to the cells pre-loaded with BAPTA. Cells were trypsinized at the end of niclosamide treatment for staining with Fluo-4, AM or processed for Annexin V-FITC staining.

Apoptotic cell death analysis by Annexin V staining

Cells were seeded at 50000 cells per well in a 6-well dish and treated with different concentrations of indicated drug compounds. At the end of the experiment total cells including both rounded floating and adherent cells were collected carefully by trypsinization and centrifuged at 400 g for 5 min. The pellet was resuspended in 200 µl of incubation buffer and centrifuged at same speed as above to collect the washed sample pellets. These pellets were then incubated with Annexin V solution premixed with the incubation buffer (10 µl of Annexin V mixed in 990 µl of incubation buffer) for 20 min in dark at room temperature. After incubation, 250 µl of incubation buffer was added to each pellet and FACS analysis performed to measure the amount of apoptotic cells. The respective apoptotic populations were determined by percentage of Alexa Fluor 488-A positive cells.

Cell-Cycle analysis

HCT116 cells with different genetic status of p53 were either DMSO treated or treated with niclosamide. Cells were collected at indicated time points to determine the cell cycle specificity. After washing with PBS, cells were transferred into 70% ethanol and incubated for 30 min. Subsequently cells were incubated with Ribonuclease A and stained with propidium iodide (PI) for 20 min and analyzed by flow cytometry.

Immunofluorescence imaging

Cells for immunofluorescence microscopy were cultured on square glass slides (22X22 mm) in 6-well plates. After treatment with niclosamide, cells were washed 3 times in PBS (phosphate buffer saline). Next, cells were incubated with ice- cold 2% formaldehyde (w/v) for 20 min and washed 3 times with PBST. Fixed cells were blocked with 5% BSA in PBST for overnight at 4ºC. After blocking; cells were stained with appropriate antibodies in PBST containing 5% BSA. Cells were washed 3 times in PBST and then co-immunostained with anti-rabbit or anti-mouse Alexa Fluor®

488 and anti-rabbit or anti-mouse Alexa Fluor® 594 for 30 min. Cells were washed 3 times with PBST and stained with DAPI. After mounting, imaging was performed with Zeiss microscope.

Cell fractionation assay

Cytosolic and mitochondrial fractionation assay was essentially performed according to manufacturer's instructions with Active Motif Mitochondrial fractionation Kit (Cat. No 40015). HCT116 cells with different p53 genetic status were grown in T175 flasks. At the end of the experiment, all cells were collected in polypropylene tube. After washing 2x in PBS (800 g at 4ºC), Cells were counted and all samples were adjusted to $2x10^7$ cell density in 400 µl of Cytosolic buffer. Cell lysate were centrifuged at 800 g for 20 min and supernatant (cytosolic + mitochondrial fraction) were collected in ice-cold micro-centrifuge tubes.

The supernatants were further centrifuged at 1000 g for 20 min to pellet mitochondria. The mitochondrial portion was washed in 1X cytosolic buffer and lysed with 100 µl of mitochondrial buffer supplemented with competing amount of Protease inhibitor and DTT. The cytosolic fractions from earlier step were centrifuged at 16000 g to remove any residual mitochondrial contamination. The protein samples were lysed in 1xSDS loading supplemented with protease inhibitor (Roche) and DTT. To determine the level of cytochrome c release in response to drug treatment, protein samples were immunoblotted with cyt c antibody.

Membrane Potential TMRE assay

HCT116 cells with different genetic status of p53 were grown in 6-well plates up to a maximum of 200000 cells per well. Cells were either DMSO treated or treated with niclosamide for different time points. Trypsinized cells were washed 2 times in PBS and incubated with TMRE dye in serum free media for 20 min. Cells were FACS sorted with red channel (peak excitation at 549 nm and emission at 575 nm)

ADP, ATP and ADP/ATP ratio measurements

Cells were seeded at $1x10^4$ cell density in 96 well plates. Next day, cells were either mock treated or treated with 1.5 µM niclosamide for 48 hrs and lysed in nucleotide releasing buffer. Then, ADP, ATP and ADP/ATP ratio were measured by using Abcam assay kit (ab65313) according to the manufacturer's protocol. Luminescence signal was measured in SpectraMax M5 Multi-Mode (Molecular Devices).

Immunohistochemistry and Fluorescent Immunostaining

Tumor tissues were fixed in 10% formalin, embedded in paraffin, sectioned at 4 µm, and stained with hematoxylin and eosin (H&E) or the required antibodies. Briefly, for antigen retrieval, sections were boiled for 20 min in Tris-EDTA buffer (pH 9.0) for staining with anti CD31 antibody

(ab28364, Abcam) and anti p53, and in citrate buffer (pH 6.0) for anti-Ki-67 (ab15580, Abcam) and anti-Cleaved Caspase-3 (9661, Cell Signaling). Antibodies were used at 1:50.1:100, 1:500 and 1:300 respectively. Images were captured using Leica SCN 400 slide scanner.

Western Blotting

Cells were lysed on ice in RIPA buffer supplemented with competing amount of protease inhibitor cocktail (Roche). Cell lysates were then sonicated for 15 sec at 10% amplitude (Qsonica- Q700 Sonicator, Misonix) and then centrifuged at $4^{o}C$ for 3 min. Clarified Protein extracts were quantified by Pierce BCA protein assay kit (Thermo Scientific). Cell lysates were mixed in 4x LDS Buffer (Invitrogen) and DTT was added. Equal amount of proteins extracts were resolved in SDS-PAGE and transferred either into nitrocellulose or into PVDF membrane. Membranes were probed with listed primary and secondary antibodies and developed by using ECL on X-Ray film.

Mitochondrial cellular respiration (Cell Mito Stress)

Cells were plated at $4x10^{5}$ cells per well into XF96-well cell culture plate in order to get a cell monolayer. Next day, cell medium was replaced with XF Base medium (pH 7.4), supplemented with 2 mM pyruvate and 20 mM glucose. Mitochondrial respiration was directly measured by oxygen consumption rates (OCR), which was performed in XF Analyzer (Seahorse Bioscience). OCR was measured with 2 μM oligomycin, 0.5 μM FCCP, and 0.5 μM antimycin A and were injected into the ports of the XF assay cartridge. In some experiments, FCCP was replaced with 3 μM of niclosamide.

Bioinformatics

Raw data (fastq files) for RNA-Seq in TCGA cohorts were obtained from the controlled-access repository hosted at dbGaP (September 2016). Raw reads were aligned to the hg19 reference genome by using *STAR* aligner [54] (https://github.com/alexdobin/STAR) with default parameters. After the alignment, *featureCounts* program [55] (http://bioinf.wehi.edu.au/featureCounts/) was used with default parameters to count the mapped reads to the genes, which were then normalized across the samples within each cancer type based on the total numbers of mapped reads.

To identify the samples with high and low expression in each cohort, known TP53 targets (13 genes) were considered together to assign a single score to each sample. First, *z*-transformation was performed for each gene based on the normalized read counts. Then for a sample, the average *z*-score across 13 genes was used to rank the sample. Based on the score, the upper and lower quartiles of the samples were regarded as the high and low expression groups,

respectively. For the heatmaps, log_2-transformation was done for the normalized read counts followed by subtraction of the mean across the samples for each gene.

Chromatin Immunoprecipitation (ChIP)

Cells were cross-linked by 1% formaldehyde and quenched using 0.125 M glycine. Lysis and sonication of the cross-linked pellets were performed to obtain ~500bp-1000 bp chromatin fragments. Supernatant was pre-cleared with 100 µl freshly prepared Protein G Sepharose beads (Sigma Aldrich, 17-5280-01) and pre-cleared supernatant was incubated with p53 DO-1 (Santa Cruz) antibody at 4°C overnight. Antibody was captured using Protein G Sepharose beads and beads were washed twice in a low salt wash buffer (20 mM Tris, pH 8.1, 2 mM EDTA, 50 mM NaCl, 1% Triton X-100 and 0.1% SDS), followed by a high salt wash buffer (10 mM Tris, pH 8.1, 1 mM EDTA, 0.25 M LiCl, 1% NP-40 and 1% deoxycholic acid) and finally a LiCl wash buffer. DNA was eluted using 250 µl 100 mM sodium bicarbonate and 1% SDS at room temperature for 15 min. Formaldehyde cross-links were reversed at 65°C with 10 µl 5 M NaCl. Samples were then treated with proteinase K for 1 hr and purified using PCR purification kit (Qiagen, 28106). QPCR was performed on the purified DNA samples using SYBR Green PCR Master Mix (Biorad, 172-5271).

Quantitative real-time PCR assays

For quantitative real time PCR analyses, total RNA was extracted from mouse kidney or spleen tissue lysates in TRIzol reagent (Invitrogen), using standard protocol. 5 µg of total RNA from each sample was used to generate high fidelity cDNA for quantitative PCR analyses. The cDNA was amplified by using Bio Rad SSO Advanced Universal Green SYBR mix. Real-time PCR were performed with primers sequences listed in supplementary table 5.

Genomic DNA Sequencing:

Genomic DNA extraction and PCR was essentially performed using KAPA Mouse Genotyping Kit (KK7302) as mentioned in the manufacturer's protocol from the tumors tissues extracted from human biopsy. DNA was sequenced for the exons of p53 sequence with primers listed in supplementary table 5.

Generation of mouse embryonic fibroblasts

Wild-type and p53R172H/R172H mouse embryonic fibroblasts were derived from littermates wild type mice and homozygous R172H mutant p53 transgenic mice at E13.5 respectively as previously described [56]. All cells were routinely cultured in Dulbecco's modified Eagle's medium with 10% fetal bovine serum and Pen-Strep.

Ovarian patient-derived xenografts and HCT116 tumor xenografts

The tumor samples used for generating the ovarian xenografts was established as per the standard protocol. Clinical data of the patient-derived tumors are in Supplementary Table 3(i) and 3(ii). In brief the tumor tissue was collected in cold, sterile RPMI 1640 medium consisting of cocktail of antibiotics, Penicillin/streptomycin (100 U/ml; 100 µg/ml) and fungizone (1 µg/ml)(all from Life Technologies). One representative portion of the tumor sample was fixed in 10% Neutral Buffered Formalin (10% NBF) and used for routine histopathological diagnosis and the other portion was utilized for xenotransplantation. The tumor tissue was then implanted subcutaneously into 4- to 6-week-old female NOD/SCID mice to create the first generation (F1) PDX mice. The tumors were monitored regularly and grown to 500 mm^3. The mice were then sacrificed and the tumors were harvested. Representative portion of PDX tumors were immediately fresh frozen, formalin fixed, stored in 90% FBS and 10% DMSO, or placed in RPMI 160 medium for further serial implantation to create subsequent generations of PDX models (F2, F3, F4, etc.). To evaluate the maintenance of the morphology and main characteristics of the tumor of origin, FFPE tissues sections from patient tumor samples and xenografts of all established PDX models were stained with H&E. In addition, these sections were immunostained to determine the expression of various markers and reviewed by a clinical pathologist.

For the efficacy studies, tumor fragments (approximately ~40 mg, F3) were implanted s.c. onto the flank of 4- to 6-week-old female NOD/SCID mice. PDX tumors were allowed to grow about 150 to 250 mm^3 in size. The animals were randomized into 2 groups (n = 10): Group-1 vehicle control (n=10) (70% PEG400, 20% D5W, 10% ethanol, p.o), and Group 2: Niclosamide (dose: 75 mg/kg, b.i.d x 28, p.o.).

For HCT116 tumor studies, NOD/SCID mice were inoculated with HCT116 $p53^{+/+}$ cells (2.5 × 10^6 cells), HCT116 $p53^{-/-}$ cells (10 × 10^6 cells) and on day 6 post implantation, the animals were randomized into 2 groups for treatment (n = 10): Group-1 vehicle control (n=10) (85% PEG 300, 15% n-methyl pyrrlodinone p.o), and Group 2: Niclosamide (dose: 100 mg/kg, b.i.d x 28, p.o.).

Body weight was measured every day and tumor growth was monitored twice weekly using a Vernier caliper. Tumor volume was calculated using the following formula $V = a \times b^2 \times 0.52$, where "a" is the largest and "b" the smallest diameter of the tumor. At the end of the treatment period, all animals were sacrificed and tumors were harvested, weighed, and observed for gross pathology. The tumors were further processed for RNA, protein and histological analysis.

Supplementary figure 1

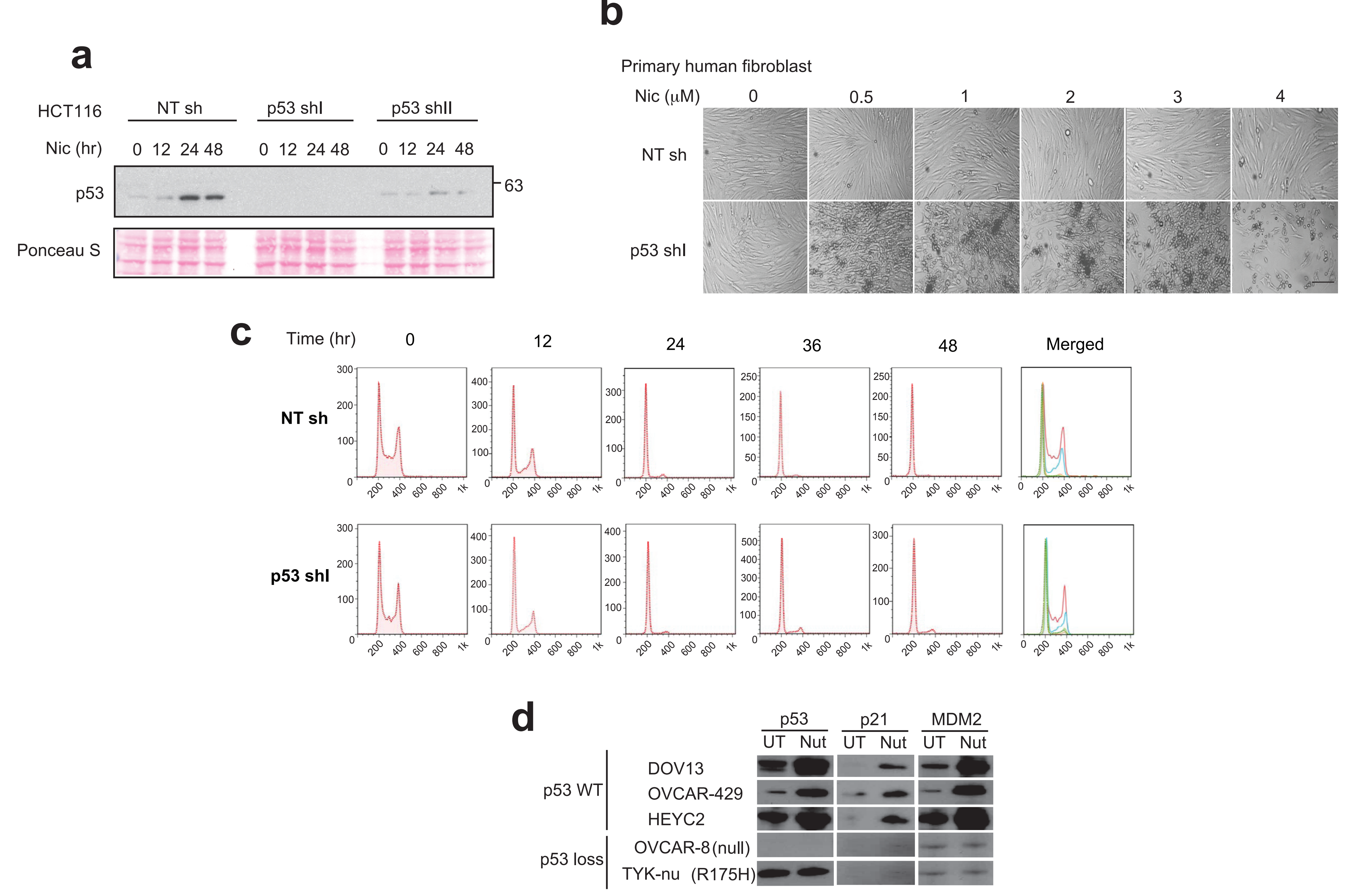

Supplementary figure 2

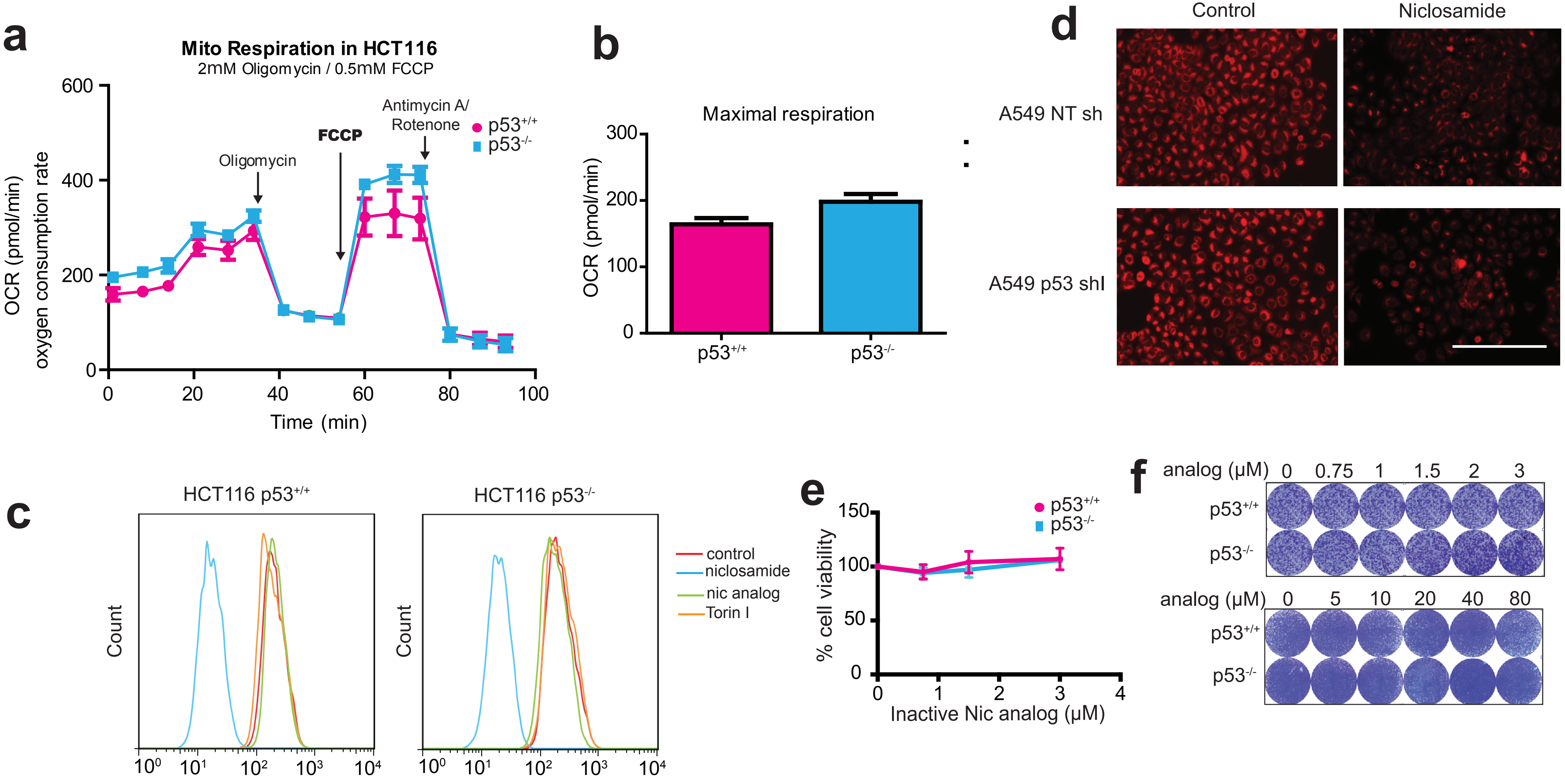

Supplementary figure 3

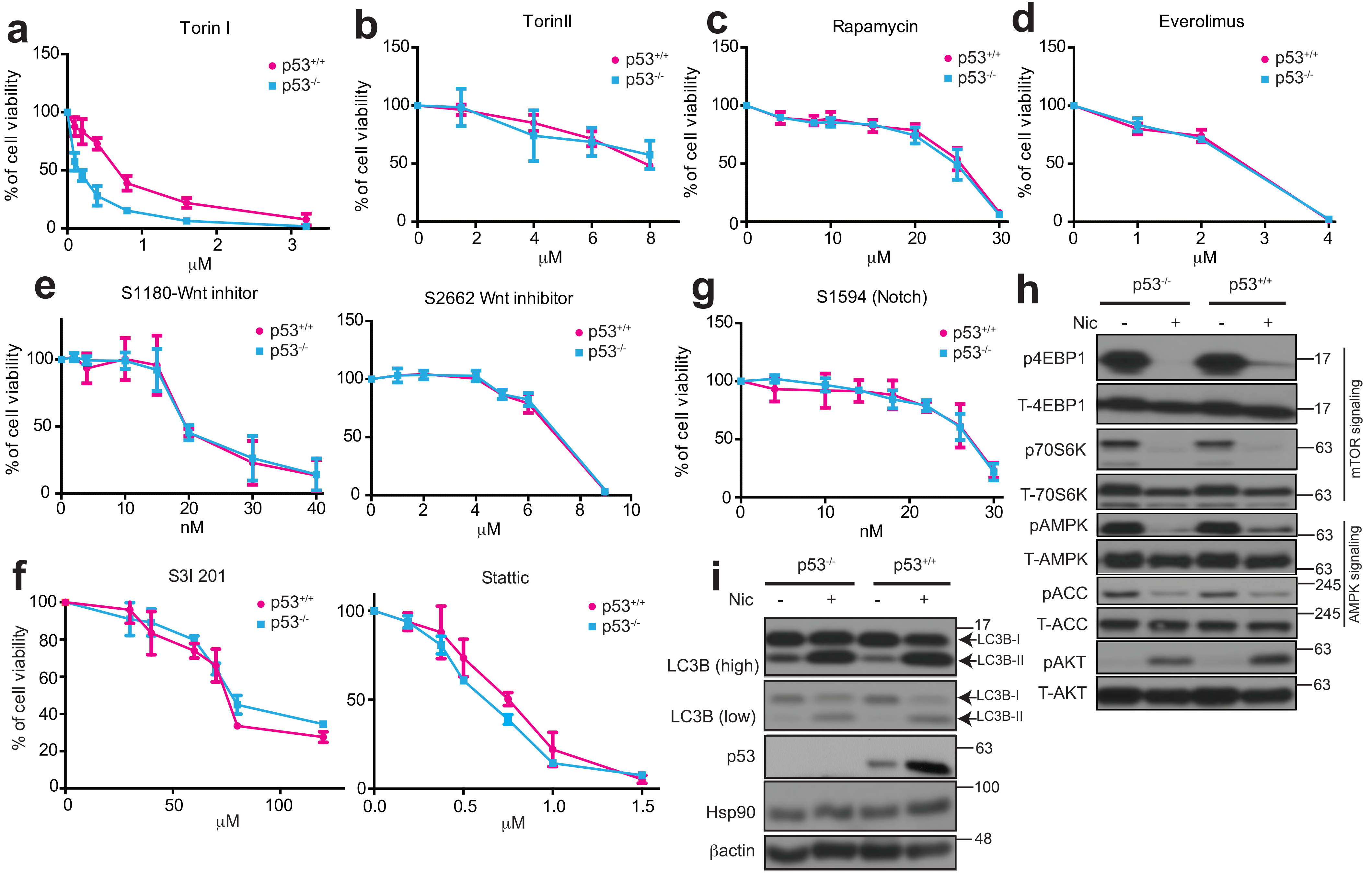

Supplementary figure 4

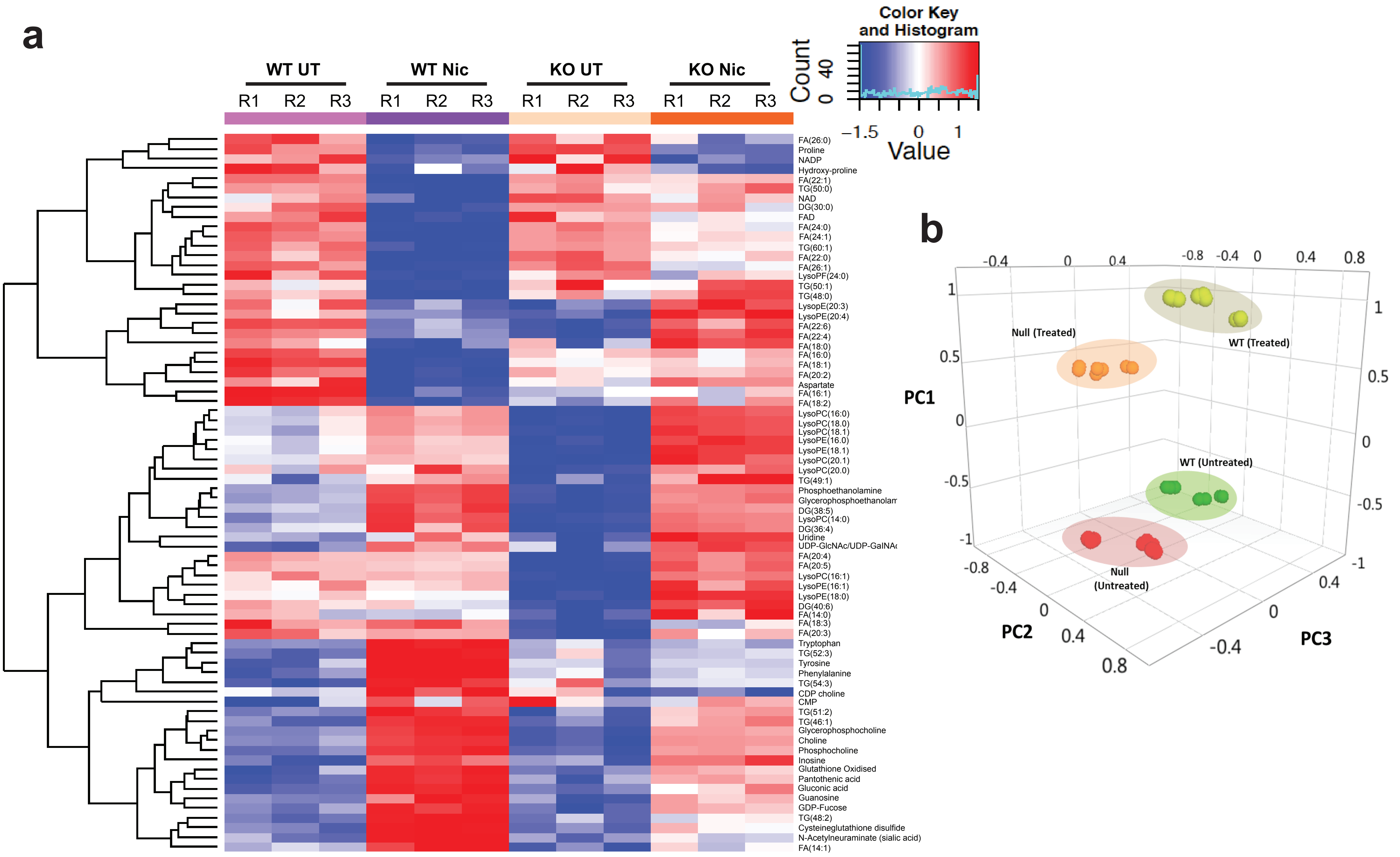

c

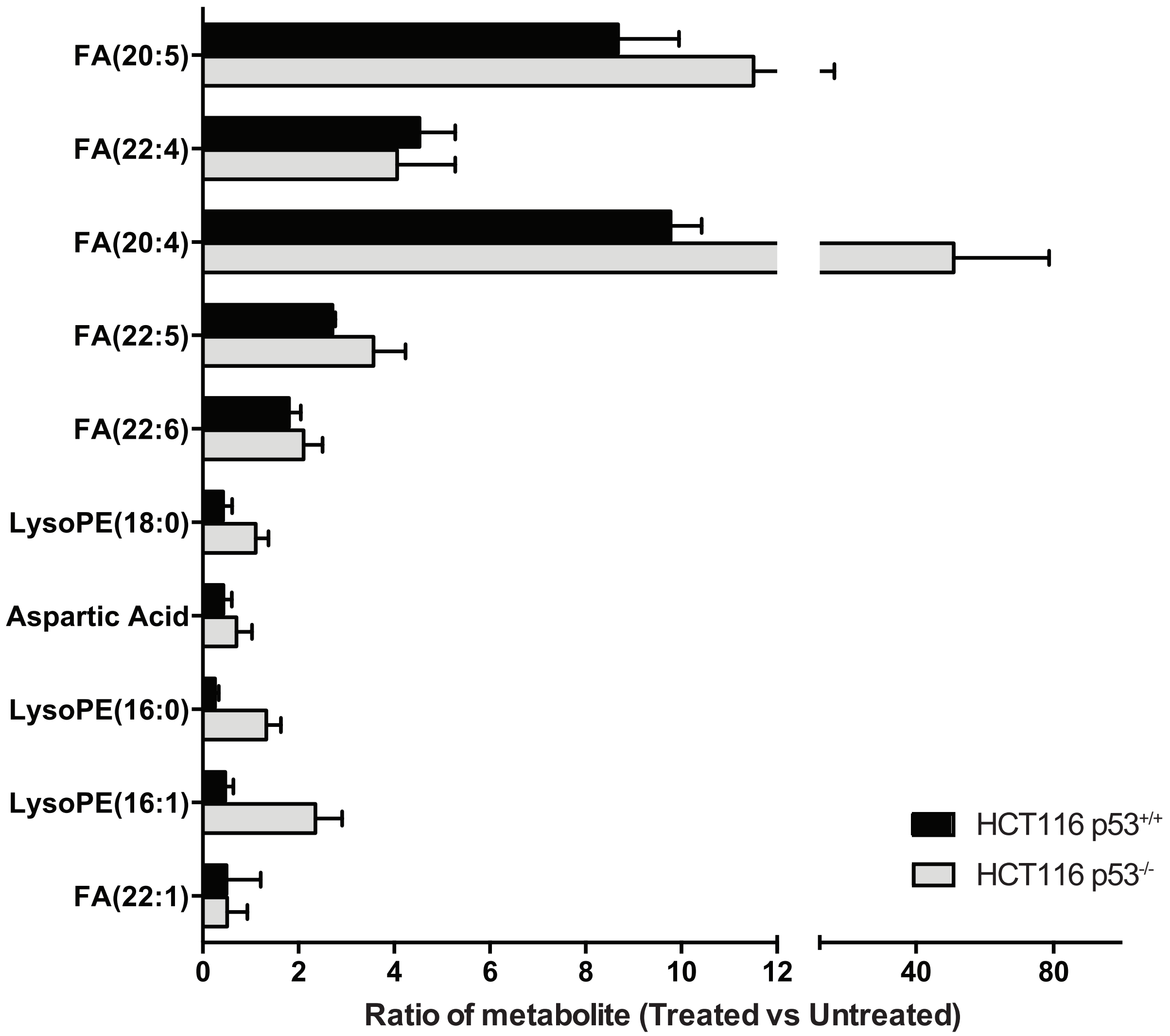

FA(20:5)
FA(22:4)
FA(20:4)
FA(22:5)
FA(22:6)
LysoPE(18:0)
Aspartic Acid
LysoPE(16:0)
LysoPE(16:1)
FA(22:1)
HCT116 p53+/+
HCT116 p53-/-
0
2
4
6
8
10
12
40
80
Ratio of metabolite (Treated vs Untreated)

Supplementary figure 5

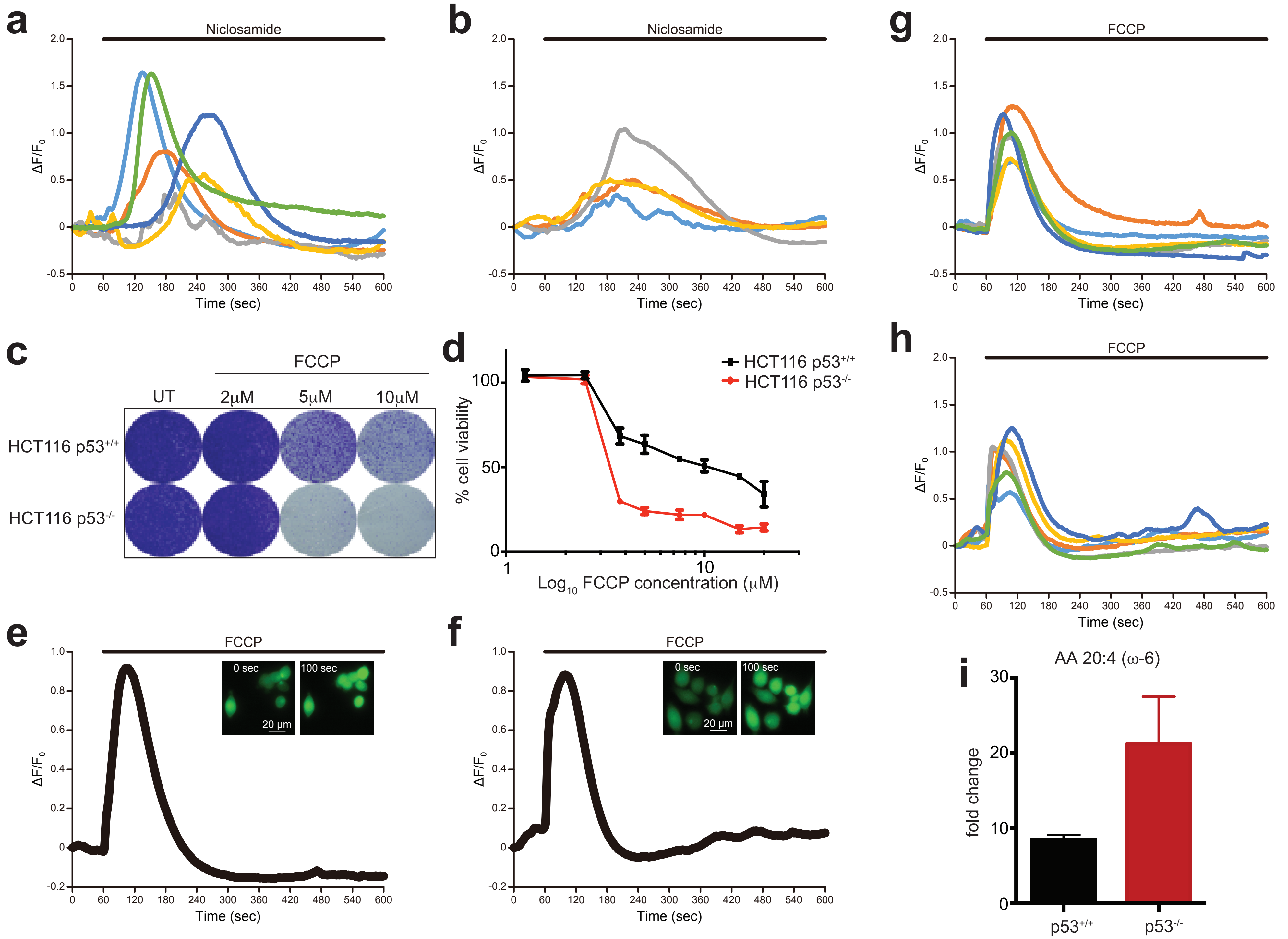

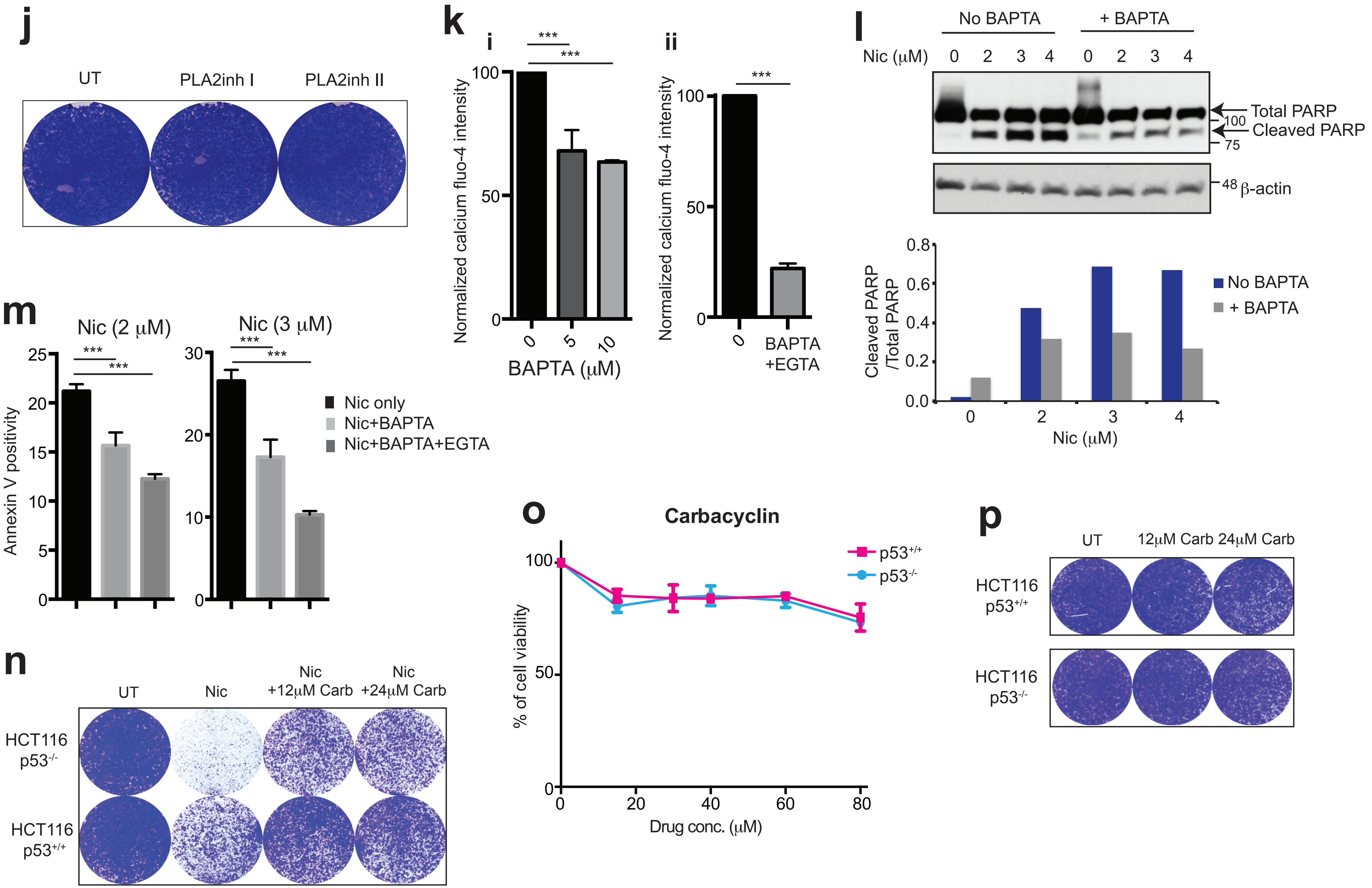

j
UT
PLA2inh I
PLA2inh II
k
i
***
***
Normalized calcium fluo-4 intensity
100
50
0
0
5
10
BAPTA (μM)
ii
***
Normalized calcium fluo-4 intensity
100
50
0
0
BAPTA
+EGTA
l
No BAPTA
+ BAPTA
Nic (μM)
0 2 3 4 0 2 3 4
Total PARP
100
Cleaved PARP
75
48
β-actin
Cleaved PARP
/Total PARP
0.8
0.6
0.4
0.2
0.0
0
2
3
4
Nic (μM)
No BAPTA
+ BAPTA
m
Nic (2 μM)
Nic (3 μM)
***
***
***
***
Annexin V positivity
25
20
15
10
5
0
30
20
10
0
Nic only
Nic+BAPTA
Nic+BAPTA+EGTA
n
UT
Nic
Nic
+12μM Carb
Nic
+24μM Carb
HCT116
p53-/-
HCT116
p53+/+
o
Carbacyclin
p53+/+
p53-/-
% of cell viability
100
50
0
0
20
40
60
80
Drug conc. (μM)
p
UT
12μM Carb
24μM Carb
HCT116
p53+/+
HCT116
p53-/-

Supplementary figure 6

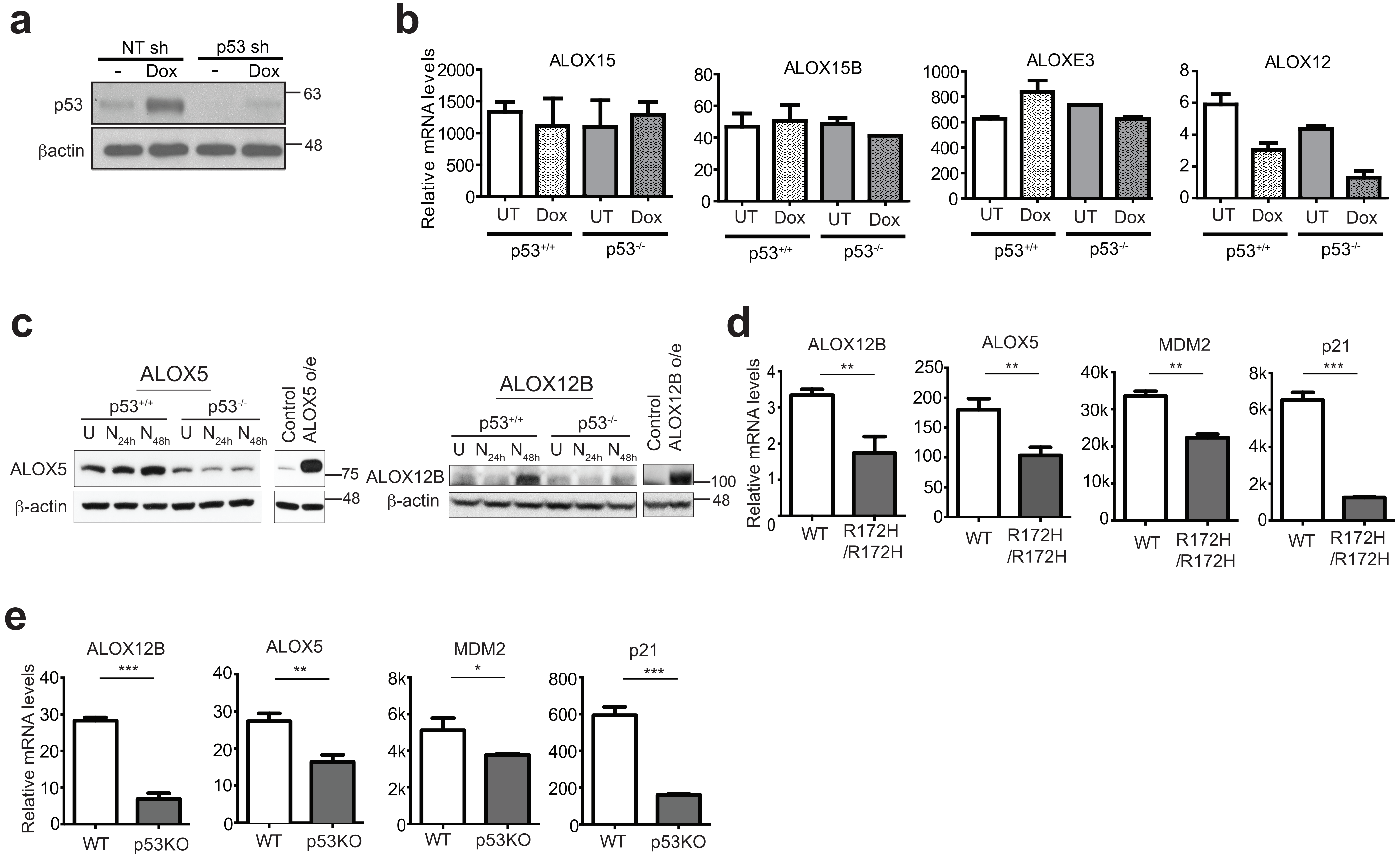

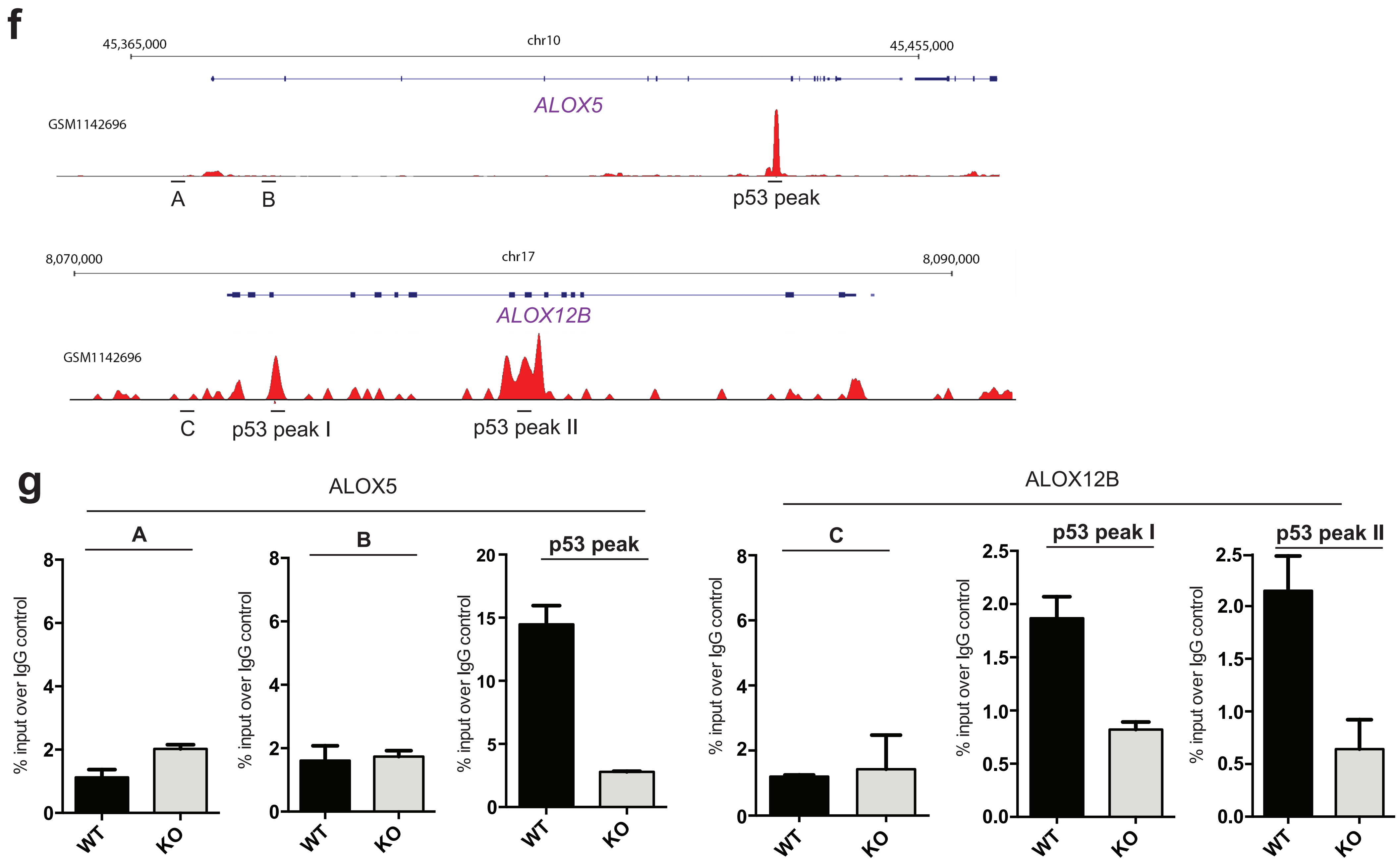
f
45,365,000
chr10
45,455,000
ALOX5
GSM1142696
A
B
p53 peak
8,070,000
chr17
8,090,000
ALOX12B
GSM1142696
C
p53 peak I
p53 peak II
g
ALOX5
ALOX12B
A
B
p53 peak
C
p53 peak I
p53 peak II
% input over IgG control
WT
KO
0
2
4
6
8
5
10
15
20
0.0
0.5
1.0
1.5
2.0
2.5

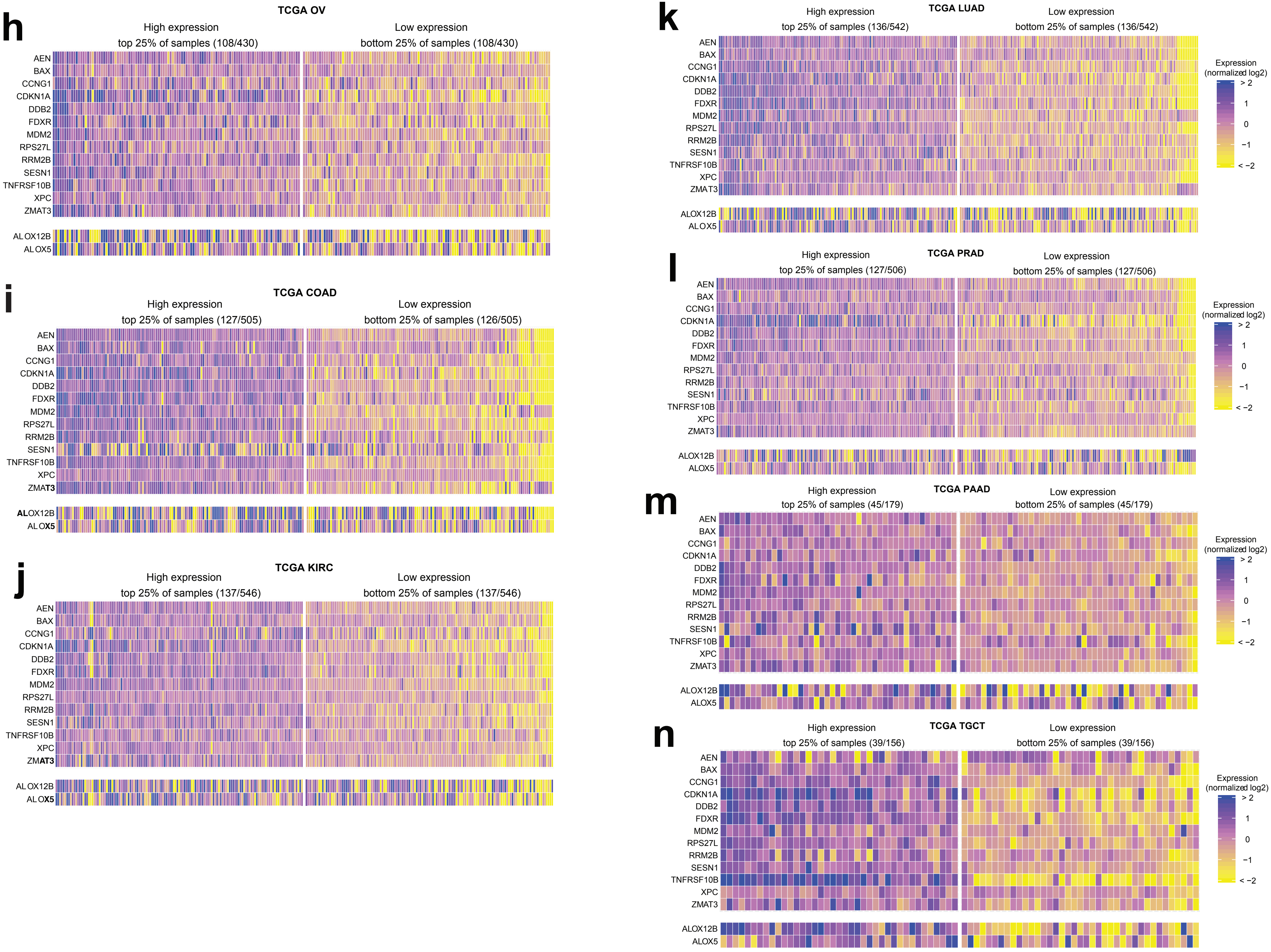
h
TCGA OV
High expression
top 25% of samples (108/430)
Low expression
bottom 25% of samples (108/430)
AEN
BAX
CCNG1
CDKN1A
DDB2
FDXR
MDM2
RPS27L
RRM2B
SESN1
TNFRSF10B
XPC
ZMAT3
ALOX12B
ALOX5
i
TCGA COAD
High expression
top 25% of samples (127/505)
Low expression
bottom 25% of samples (126/505)
AEN
BAX
CCNG1
CDKN1A
DDB2
FDXR
MDM2
RPS27L
RRM2B
SESN1
TNFRSF10B
XPC
ZMAT3
ALOX12B
ALOX5
j
TCGA KIRC
High expression
top 25% of samples (137/546)
Low expression
bottom 25% of samples (137/546)
AEN
BAX
CCNG1
CDKN1A
DDB2
FDXR
MDM2
RPS27L
RRM2B
SESN1
TNFRSF10B
XPC
ZMAT3
ALOX12B
ALOX5
k
TCGA LUAD
High expression
top 25% of samples (136/542)
Low expression
bottom 25% of samples (136/542)
AEN
BAX
CCNG1
CDKN1A
DDB2
FDXR
MDM2
RPS27L
RRM2B
SESN1
TNFRSF10B
XPC
ZMAT3
ALOX12B
ALOX5
Expression
(normalized log2)
> 2
1
0
−1
< −2
l
TCGA PRAD
High expression
top 25% of samples (127/506)
Low expression
bottom 25% of samples (127/506)
AEN
BAX
CCNG1
CDKN1A
DDB2
FDXR
MDM2
RPS27L
RRM2B
SESN1
TNFRSF10B
XPC
ZMAT3
ALOX12B
ALOX5
Expression
(normalized log2)
> 2
1
0
−1
< −2
m
TCGA PAAD
High expression
top 25% of samples (45/179)
Low expression
bottom 25% of samples (45/179)
AEN
BAX
CCNG1
CDKN1A
DDB2
FDXR
MDM2
RPS27L
RRM2B
SESN1
TNFRSF10B
XPC
ZMAT3
ALOX12B
ALOX5
Expression
(normalized log2)
> 2
1
0
−1
< −2
n
TCGA TGCT
High expression
top 25% of samples (39/156)
Low expression
bottom 25% of samples (39/156)
AEN
BAX
CCNG1
CDKN1A
DDB2
FDXR
MDM2
RPS27L
RRM2B
SESN1
TNFRSF10B
XPC
ZMAT3
ALOX12B
ALOX5
Expression
(normalized log2)
> 2
1
0
−1
< −2

Supplementary figure 7

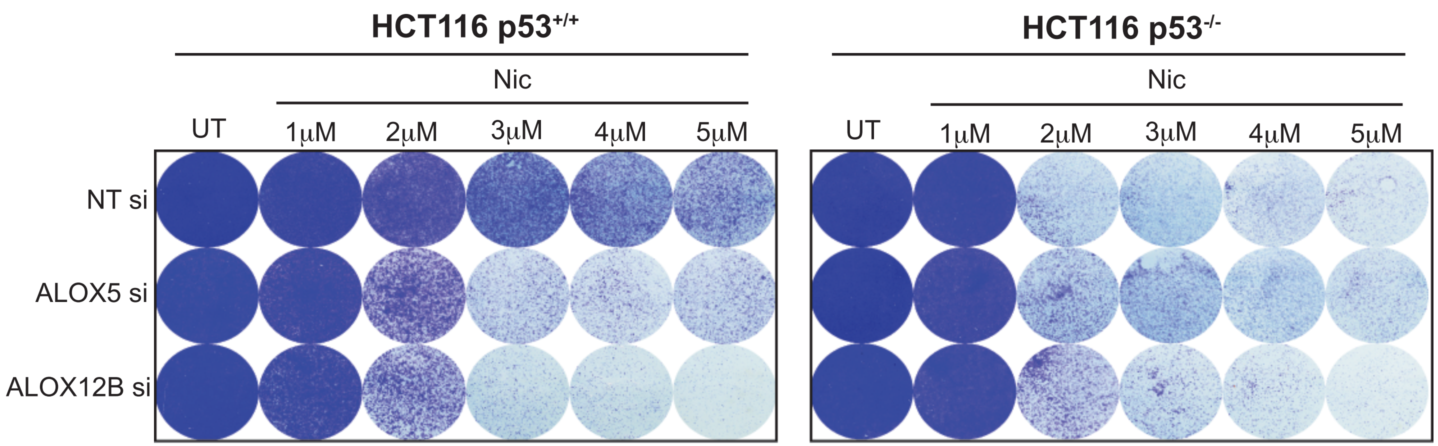

Supplementary figure 8

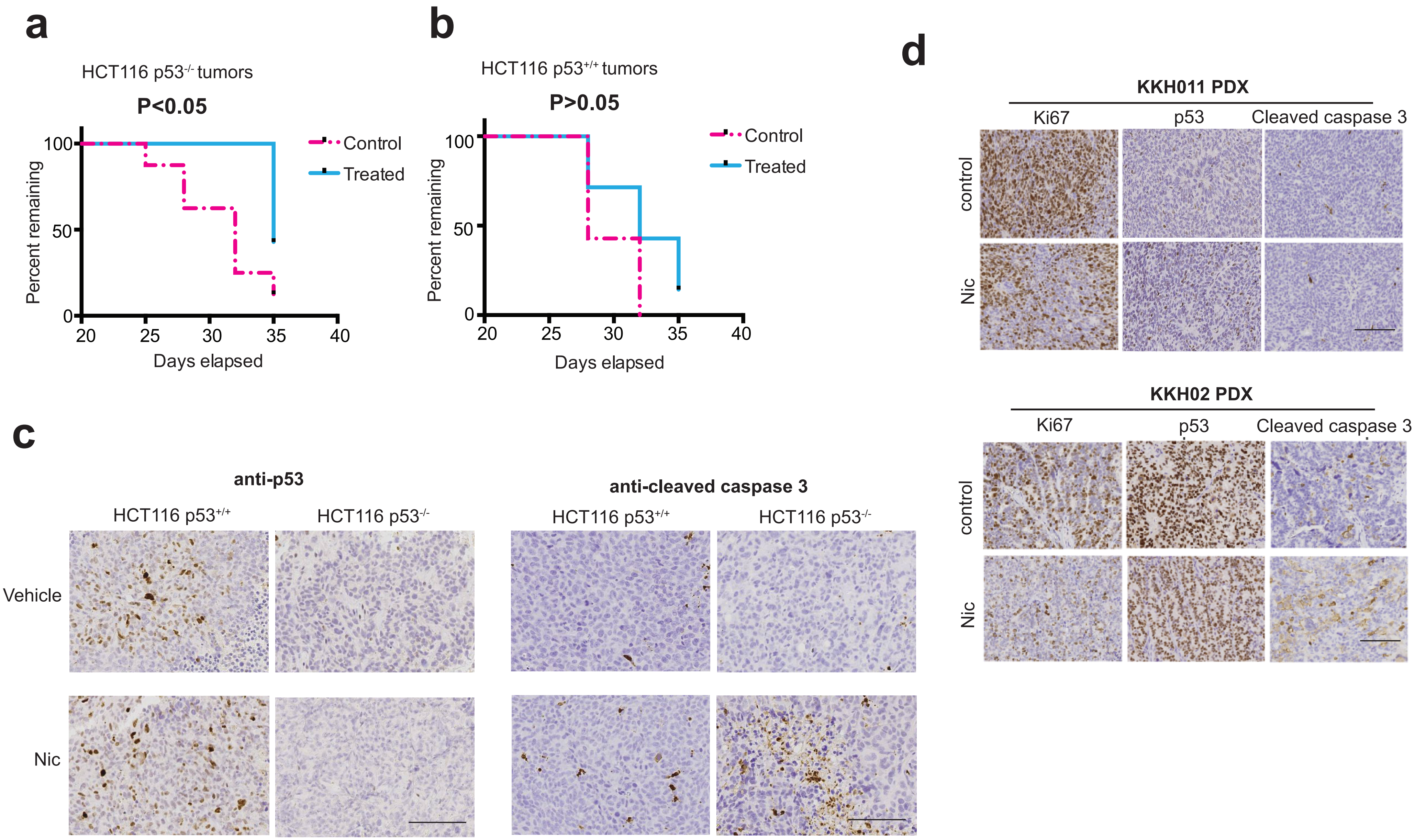

Supplement: Supplementary file 1 — Supplementary Information [file 41467_2018_5805_MOESM1_ESM.pdf]
