# Supplementary material for: Mitochondrial uncoupling reveals a novel therapeutic opportunity for p53-defective cancers
Source: Nat Commun. 2018 Sep 26;9:3931. doi: 10.1038/s41467-018-05805-1 (PMC6158291; doi:10.1038/s41467-018-05805-1)
Supplement: Supplementary file 3 — Description of Additional Supplementary Files [file 41467_2018_5805_MOESM3_ESM.pdf]

Description of Additional Supplementary Files

File Name: Supplementary Movie 1
Description: Niclosamide induces calcium burst in HCT116 p53-/- cells.

File Name: Supplementary Movie 2
Description: Niclosamide analog does not induce calcium burst in HCT116 p53-/-.

File Name: Supplementary Movie 3
Description: FCCP induces calcium burst in HCT116 p53-/- cells.

File Name: Supplementary Movie 4
Description: DMSO (solvent) does not induce calcium burst in HCT116 p53-/-cells.

File Name: Supplementary Movie 5
Description: Niclosamide induces calcium burst in HCT116 p53+/+ cells.

File Name: Supplementary Movie 6
Description: Niclosamide analog does not induce calcium burst in HCT116 p53+/+.

File Name: Supplementary Movie 7
Description: FCCP induces calcium burst in HCT116 p53+/+ cells.

File Name: Supplementary Movie 8
Description: DMSO (solvent) does not induce calcium burst in HCT116 p53+/+cells.
